# Different Mechanisms of Two Subtypes of Perforating Artery Infarct in the Middle Cerebral Artery Territory: A High-Resolution Magnetic Resonance Imaging Study

**DOI:** 10.3389/fneur.2018.00657

**Published:** 2018-09-20

**Authors:** Siyuan Liao, Zhezhi Deng, Yuge Wang, Ting Jiang, Zhuang Kang, Sha Tan, Yilong Shan, Yan Zou, Zhengqi Lu

**Affiliations:** ^1^Department of Neurology, Third Affiliated Hospital of Sun Yat-sen University, Guangzhou, China; ^2^Department of Radiology, Third Affiliated Hospital of Sun Yat-sen University, Guangzhou, China

**Keywords:** perforated artery disease, branch atheromatous disease, lacunar infarct, high-resolution MRI, artery remodeling

## Abstract

**Purpose:** Perforating Artery Infarcts (PAIs) can be divided into two subtypes based on their etiologies: branch Atheromatous Disease (BAD) and Lacunar Infarct (LI). Recent studies have shown that while both subtypes can be caused by large artery lesions, the different mechanisms that underlie their development are not clear. This study was designed to use High-Resolution Magnetic Resonance Imaging (HRMRI) to explore the differences that contribute to the occurrence of these two subtypes in large artery lesions in the anterior circulation.

**Methods:** Fifty patients with an acute PAI in the anterior circulation were enrolled (32 BAD and 18 LI patients). The ipsilateral middle cerebral artery (MCA) was scanned with HRMRI to analyze the atherosclerosis plaques. Artery remodeling and plaque characteristics of MCA lesions were compared between the two subtypes.

**Results:** The rate of MCA lesions was significantly higher in BAD and substantially lower in LI (*P* = 0.033). LAs for the lumen areas in Bad, they were smaller than LI (*P* < 0.001), Additionally, the plaque area (*P* = 0.001) and plaque burden (*P* < 0.001) were superior in the BAD group. Most BAD patients displayed non-positive remodeling, while the great majority of LI patients showed positive remodeling (*P* < 0.001).

**Conclusion:** In the anterior circulation, a considerable amount of BAD and LI share similarities with atherosclerotic plaques in large arteries. BAD patients mainly showed relatively large and stable atherosclerotic plaques in large arteries, while LI patients mainly exhibited relatively small and unstable atherosclerotic plaques.

**Clinical Trial Registration:** This clinical trial is a retrospective study and therefore does not require registration.

## Introduction

**Figure 1:**
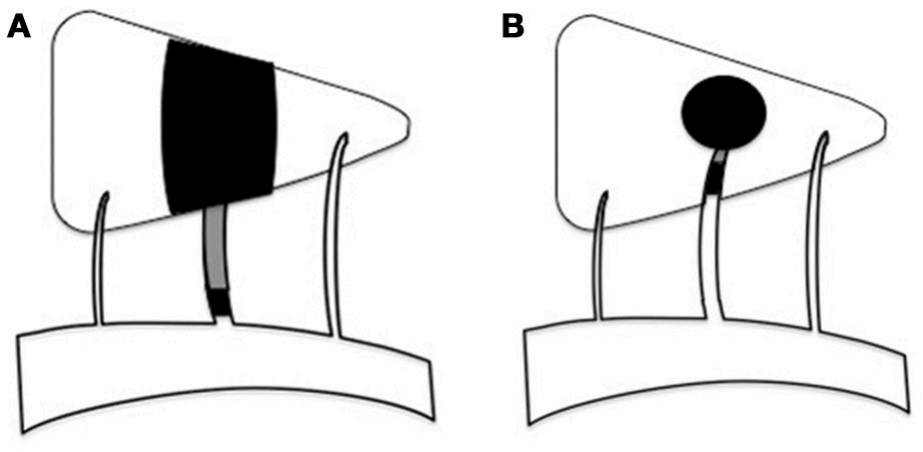
Schematic diagram of BAD and LI in anterior circulation. **(A)** BAD is considered to be caused by large artery atherosclerosis obstruction proximal branch or orifice of the penetrating artery. **(B)** LI is described as lipid hyalinization at the distal end of the perforating artery.

Perforating artery infarcts (PAIs) occurring in the middle cerebral artery (MCA) territory play an important role in ischemic stroke, especially in Asian populations (1). PAIs are classified into several subtypes based on differences in their etiologies, including branch atheromatous disease (BAD) and lacunar infarct (LI). BAD is thought to be caused when atherosclerosis in a large artery obstructs a proximal branch or orifice of the penetrating artery (2) (Figure 1A). BAD is assigned to both large and small artery atherosclerosis in the TOAST classification system (3). LI is caused by small vessel occlusion and is described as lipid hyalinization at the distal end of the perforating artery (4) (Figure 1B). However, recent studies have shown that LI with atherosclerotic lesions in large arteries in the anterior circulation (5, 6) have an incidence as high as 46% (7). This is higher than the incidence of asymptomatic atherosclerosis, suggesting that LI is not limited to small artery lesions, many of which can be closely related to upstream arterial lesions (8). While BAD and LI may both be associated with large artery atherosclerosis, these two subtypes of PAIs are quite different. However, the mechanisms underlying these differences are not clear.

An increasing number of studies show that vascular morphology and plaque composition are better indicators than the rate of stenosis for distinguishing different ischemic stroke mechanisms and assessing risks (9–11). By analyzing artery remodeling patterns, plaque size, and the vulnerabilities of different lesions, we can explore the differences in pathogenesis and disease process underlying these conditions. High-resolution magnetic resonance imaging (HRMRI) is a noninvasive technique that assesses artery wall morphology and atherosclerosis plaque characteristics by obtaining morphological measurements and assessing plaque composition and stability (12, 13). In addition, HRMRI produces acceptably reproducible results for plaque measurements and vessel quantification (14). Therefore, HRMRI-based MCA analyses can reveal the structure of an artery wall and the composition of plaques, both of which are helpful metrics for exploring the mechanisms underlying stroke and selecting targeted preventive measures. Thus, we used HRMRI to investigate the remodeling mode and distinct features of the MCA wall morphology in patients with isolated infarcts in the basal ganglia to identify differences in the mechanisms underlying BAD and LI.

## Materials and methods

### General information

This was a single-center cross-sectional study based on data obtained from patients in the Third Affiliated Hospital of Sun Yat-sen University. The study was approved by the medical ethics committee of the Third Affiliated Hospital of Sun Yat-sen University. From October 2015 to February 2017, we recruited consecutive patients who were admitted to the hospital for anterior circulation perforated artery regional acute ischemic stroke within 7 days of stroke onset. The inclusion criteria included the following: (1) age ≥ 18 years old, (2) underwent HRMRI, (3) diagnosed as acute PAI in the anterior circulation on the basis of clinical neurological deficit symptoms and diffusion-weighted imaging (DWI), and (4) signed an informed consent form. Patients were excluded if they (1) had a history of stroke in the ipsilateral anterior circulation; (2) had ≥50% stenosis in the ipsilateral MCA or internal carotid on magnetic resonance angiography (MRA) or Doppler ultrasound (3, 15); (3) had another artery stenosis mechanism, such as moyamoya disease, dissection or vacuities; (4) showed evidence of potential sources of cardioembolism; or (5) received rt-PA thrombolytic therapy after onset or (6) if incomplete clinical data were available or poor imaging quality prevented us from analyzing the results.

**Figure 2:**
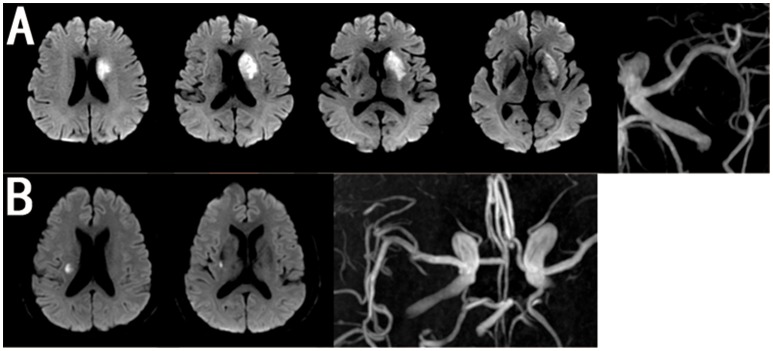
Representative MRI Images of BAD and LI in MCA. **(A)** BAD in anterior circulation with MRA showing none moderate or severe stenosis in ipsilateral MCA. **(B)** LI in anterior circulation with MRA showing none moderate or severe stenosis in ipsilateral MCA.

We divided the patients into two groups according to the lesion location and size on DWI as follows: BAD in the blood supply region of the lenticulostriate arteries (LSA) was defined as an infarction lesion with a diameter ≥15 mm that appeared in ≥3 axial slices on DWI (16), while LI was defined as an infarction lesion in the LSA region with a diameter < 15 mm and that appeared in no more than two axial slices (16) (Figure 2).

Baseline and clinical information were systematically collected and included age, gender, risk factors, and neurological symptom scores [Institutes of Health Stroke Scale (NIHSS) scores on admission and at discharge, Modified Rankin Scale (mRS) scores on admission and at discharge, and progression (defined as a worsening by ≥1 point on the NIHSS during the first 2 days)]. Patients were required to complete a laboratory examination and to undergo cardiac tests (electrocardiography and echocardiography), cervical vascular ultrasonography and magnetic resonance imaging (MRA, DWI, and HRMRI).

### MRI parameters

All patients underwent MRI on a 3-Tesla scanner (GE Discovery MR750) with a standard 8-channel head coil. The MRI protocol was as follows: (1) 3-dimensional time-of-flight MRA (repetition time (TR)/echo time (TE) = 25/3.4 ms, flip angle = 20°, field-of-view (FOV) = 20 × 16 cm, matrix size = 380 × 320, slice thickness = 0.8 mm, number of excitations (NEX) = 1, (2) axial and sagittal DWI (TR/TE = 2300/65.4 ms, FOV = 24 × 24 cm, matrix size = 160 × 160, slice thickness = 5 mm, NEX = 2), (3) fat-suppressed fast spin echo T2-weighted images (fs FSE T2WI) (TR/TE = 3200/50.8 ms, FOV = 13 × 13 cm, matrix size = 256 × 256, slice thickness = 2 mm, NEX = 4, scan pixels = 0.5 × 0.5 mm, reconstructed pixels = 0.25 × 0.25 mm), and (4) HRMRI T1WI and enhancing sequence (TR/TE = 500/18.5 ms, FOV = 20.4 × 18.4 cm, matrix size = 320 × 256, slice thickness = 0.8 mm, NEX = 2, scan volume pixels = 0.64 × 0.8 × 0.8 mm, reconstructed volume pixels = 0.8 × 0.8 × 0.8 mm) using Gadovist (1 mmol/kg) as the MRI contrast agent and starting the enhancing sequence scan at the end of the intravenous injection.

### Image analysis

All MR images were reviewed and analyzed on a workstation (GE advantage workstation AW4.6). All artery wall measurements were performed on fs FSE T2WI sequence. All images were measured by two independent readers who were blinded to the clinical materials. The mean values of quantitative data between them were applied to final analysis. When their qualitative data were contradictory, a discussion involved a third reader, who decided the final result.

**Figure 3:**
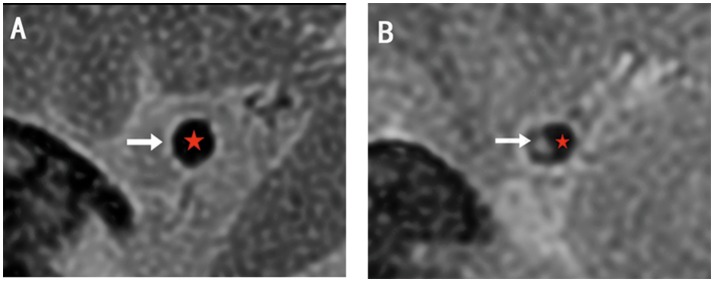
The examples of HRMRI T2WI. **(A)** The normal lumen shows vessel wall with high signal (white arrow) and smooth lumen with low signal (red star). **(B)** The lesion site shows plaque with relatively high signal (white arrow) and narrow lumen (red star). The structures are clear and can be divided.

The lesion site was defined as the slice with the severest stenosis lumen of MCA. The two reference sites were slices just distal and proximal to the lesion site that showed no atherosclerotic plaque in the artery wall (Figure 3). The mean value of the two sites was applied in the final analysis. All sites were selected by visual comparison.

**Figure 4:**
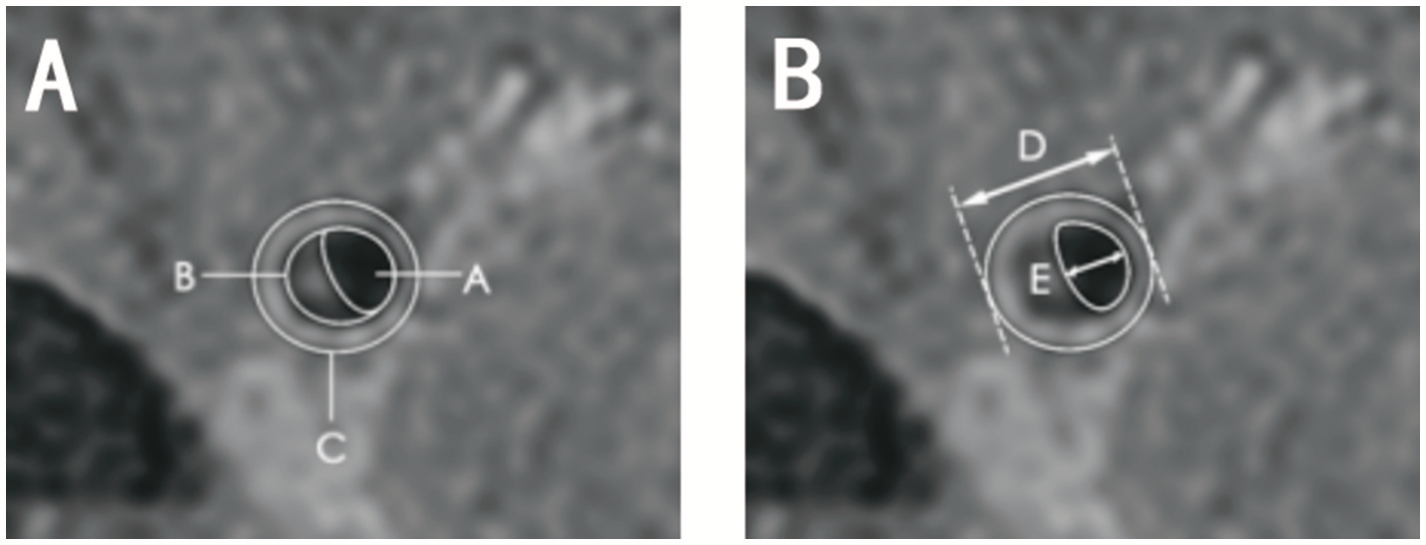
Diagram of quantitative measurements on HRMRI T2WI. **(A)** Area A, lumen area; area B, plaque area; area C, vessel area; C-A, wall area; **(B)** length D, vessel diameter; length E, lumen diameter, D-E, wall thickness.

Vessel area (VA) was calculated automatically by examining the vessel-cerebral spinal fluid interface. Lumen area (LA) was calculated at the vessel-blood interface. We selected the slice with the thickest plaque to measure lumen diameter. The following equations were used: wall area (WA) = vessel area-LA, plaque area = WA at the lesion site-WA at the reference site (Figure 4A), wall thickness = vessel diameter-lumen diameter (Figure 4B), wall thickness index = wall thickness at the lesion site/reference sites, wall area index = WA at the lesion site/reference sites, plaque burden = (plaque area/vessel area at the lesion site) × 100%, and stenosis rate = (1-LA at the lesion site/reference site) × 100%.

The remodeling index (RI) was defined as the ratio between the vessel area at the lesion site and that at the reference site. Positive remodeling (PR) was defined as RI > 1.05, negative remodeling (NR) was defined as RI < 0.95, and intermediate remodeling was defined as an RI between 0.95 and 1.05. Negative remodeling and intermediate remodeling were combined into a non-positive remodeling (non-PR) group (17).

Eccentric plaque was defined as plaque surrounding lesion lumen < 75%. Intraplaque hemorrhage was defined as a plaque area with a signal higher than 150% of that of the adjacent white matter on an HRMRI T1WI sequence (14). Plaque enhancement was defined as a plaque area in which the signal on HRMRI T1WI enhanced imaging was higher than that on an HRMRI T1WI sequence.

### Statistical analysis

All data were analyzed in SPSS 22.0. Measurement data are expressed as the mean ± standard deviation (x ± s) and were compared with Student's *t*-test for normally distributed variables. Non-normally distributed data are shown as the median and interquartile range and were compared using the Mann-Whitney U test. Categorical data are shown as numbers and percentages (%) and were compared by the chi-square test and Fisher's exact test. All analyses were two-sided, and *P* < 0.05 indicated statistical significance.

## Results

### Proportions of MCA atherosclerosis plaque

**Figure 5:**
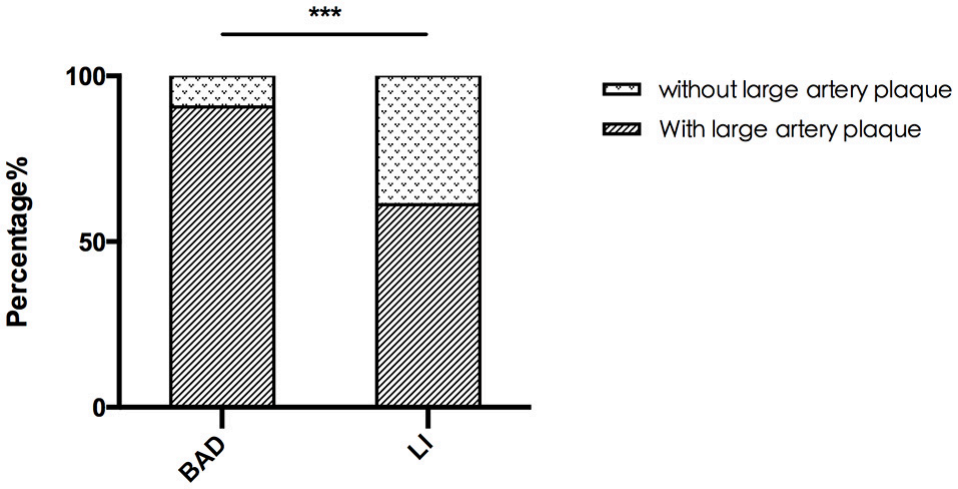
The rate of MCA lesion for BAD and LI. The rate of MCA lesion for BAD is significant higher than that for LI in anterior circulation. ^***^P < 0.05.

A total of 50 patients with acute PAI in the anterior circulation were enrolled. Of these, 32 patients were allocated to the BAD group, and 18 patients were allocated to the LI group according to DWI. We found that 29 (90.6%) of the BAD cases and 11 (61.1%) of the LI cases had ipsilateral atherosclerotic plaque in the MCA (Table 1). The rate of MCA lesions in the anterior circulation was significantly higher in the BAD group than in the LI group (Figure 5).

**Table 1:** The rate of MCA lesion for BAD is significant higher than that for LI in anterior circulation.

	**BAD (*n* = 32)**	**LI (*n* = 18)**	***P***
With plaque (%)	29(90.6%)	11(61.1%)	0.033

### General characteristics

The detailed general characteristics, including demographics, vascular risk factors, and laboratory data, of the BAD and LI groups are summarized in Tables 2, 3. We found no significant difference in traditional stroke risk factors between the two subtypes.

**Table 2:** Comparison of risk factors between BAD and LI in anterior circulation.

	**BAD (*n* = 29)**	**LI (*n* = 11)**	***P***
Age (year)	62.59 ± 13.0	61.64 ± 10.30	0.829
Male (%)	21(72.4%)	5(45.5%)	0.110
BMI	22.90 ± 2.70	24.71 ± 3.30	0.112
Hypertension (%)	18(62.1%)	9(81.8%)	0.416
Diabetes mellitus (%)	9(31.0%)	5(45.5%)	0.629
Coronary heart disease (%)	2(6.9%)	0(0.0%)	1.000
Hyperlipidemia (%)	14(48.3%)	9(81.8%)	0.119
Fatty liver (%)	2(6.9%)	3(27.3%)	0.228
Smoking (%)	8(27.6%)	2(18.2%)	0.838
Carotid arteries stenosis (%)	13(44.8%)	4(36.4%)	0.900

**Table 3:** Comparison of laboratory tests between BAD and LI in anterior circulation.

	**BAD (*n* = 29)**	**LI (*n* = 11)**	***P***
Leukocytes (10^9^/L)	7.48 ± 1.91	7.37 ± 1.59	0.871
Erythrocytes (10^12^/L)	4.61 ± 0.59	4.94 ± 0.77	0.156
Hemoglobin (g/L)	184.66 ± 263.45	135.45 ± 13.84	0.543
Platelet (10^9^/L)	227.07 ± 76.76	230.18 ± 75.26	0.909
Eosinophil granulocyte (%)	0.02 ± 0.02	0.04 ± 0.04	0.258
Fasting blood glucose (mmol/L)	6.33 ± 2.24	6.80 ± 3.85	0.631
Uric acid (umol/L)	352.56 ± 97.28	347.60 ± 119.30	0.898
Total cholesterol (mmol/L)	4.65 ± 1.07	4.94 ± 1.22	0.463
Triglyceride (mmol/L)	1.31 ± 0.45	1.57 ± 0.81	0.320
HDL-c (mmol/L)	1.05 ± 0.24	1.09 ± 0.25	0.663
LDL-c (mmol/L)	3.06 ± 0.92	3.22 ± 0.99	0.630
C-reactive protein (mg/L)	2.10(1.00–4.20)	1.50(0.65–5.20)	0.630
ESR (mm/H)	11.48 ± 8.87	15.75 ± 13.14	0.301
HbA1c (%)	6.03 ± 1.02	6.36 ± 1.63	0.439
Homocysteine (umol/L)	12.90 ± 5.22	15.06 ± 10.10	0.379
25OH-VD (nmol/L)	51.26 ± 26.39	56.33 ± 21.37	0.612

The scores for neurological symptoms and progression were similar between the two groups (Table 4). One reason for these negative statistical results may be that HRMRI requires up to 40 min to perform, and patients need to keep their body still throughout the scan. We therefore enrolled only patients with mild symptoms and excluded those with more severe symptoms, and this may have affected the statistical results.

**Table 4:** Comparison of clinical manifestations between BAD and LI in anterior circulation.

	**BAD (*n* = 29)**	**LI (*n* = 11)**	***P***
NIHSS on admission	3.00(2.00-5.00)	4.00(3.00-7.50)	0.720
mRS on admission	2.00(1.00-4.00)	3.00(1.00-4.00)	1.000
Hospitalization time (day)	12.00(8.00-16.00)	9.00(7.00-12.00)	0.128
NIHSS at discharge	2.00(1.00-5.00)	2.00(1.50-4.50)	0.656
mRS at discharge	2.00(1.00-4.00)	1.00(0.50-2.00)	0.241
ΔNIHSS score	1.00(1.00-3.00)	1.00(1.00-3.50)	0.862
ΔmRS score	0.00(0.00-1.00)	1.00(0.00-2.50)	0.213
Progression (%)	13(44.8%)	2(18.2%)	0.235

### Artery wall morphology and plaque characteristics

Vascular morphology, including vessel area, LA and wall area, was similar among stroke patients at the reference site (*P* > 0.5), indicating that the shapes of the normal walls were quite similar between the two groups even though MCA morphology showed individual and side differences. Hence, we can analyze both absolute values and ratios when comparing imaging characteristics at the lesion site between the two groups.

The differences in artery wall morphology at the lesion site and plaque characteristics between BAD and LI are distinct (Table 5). LA was smaller and wall was thicker in the BAD group than in the LI group (LA, 3.99 ± 1.29 mm^2^ vs. 6.39 ± 1.05 mm^2^, *P* < 0.001; wall thickness, 3.01 ± 0.32 mm vs. 2.59 ± 0.32 mm, *P* < 0.001). As for plaque characteristics, although most patients in the BAD and LI groups showed an eccentric plaque distribution (BAD: 89.7%, LI: 100%), plaque area and plaque burden were higher in BAD patients than in LI patients (plaque area, 2.45 ± 0.75 mm^2^ vs. 1.48 ± 0.36 mm^2^, *P* = 0.001; plaque burden, 13.41 ± 3.72% vs. 7.39 ± 1.85%, *P* < 0.001). In addition, the rate of severe stenosis was higher in the BAD group than in the LI group (43.3 ± 8.19% vs. 6.93 ± 6.84%, *P* < 0.001).

**Table 5:** Imaging data of MCA atherosclerosis for BAD and LI in anterior circulation.

	**BAD (*n* = 29)**	**LI (*n* = 11)**	***P***
**WALL MORPHOLOGY**			
**Reference site**			
Vessel area (mm^2^)	18.84 ± 3.26	18.68 ± 3.61	0.895
Lumen area (mm^2^)	6.99 ± 1.79	6.92 ± 1.50	0.911
Wall area (mm^2^)	11.85 ± 2.02	11.76 ± 2.17	0.902
Vessel diameter (mm)	4.85 ± 0.47	4.77 ± 0.51	0.609
Lumen diameter (mm)	2.92 ± 0.41	2.93 ± 0.35	0.931
Wall thickness (mm)	1.93 ± 0.27	1.83 ± 0.18	0.263
**Lesion site**			
Vessel area (mm^2^)	18.29 ± 2.99	19.62 ± 3.59	0.239
Lumen area (mm^2^)	3.99 ± 1.29	6.39 ± 1.05	< 0.001
Vessel diameter (mm)	4.84 ± 0.43	4.93 ± 0.42	0.548
Lumen diameter (mm)	1.83 ± 0.34	2.34 ± 0.30	< 0.001
Wall thickness (mm)	3.01 ± 0.32	2.59 ± 0.32	0.001
Wall area (mm^2^)	14.30 ± 2.26	13.23 ± 2.71	0.216
Wall thickness index	1.57 ± 0.19	1.41 ± 0.14	0.015
Wall area index	0.95 ± 0.12	0.97 ± 0.04	0.383
**PLAQUE CHARACTERISTICS**			
Plaque area (mm^2^)	2.45 ± 0.75	1.48 ± 0.63	0.001
Plaque burden (%)	13.41 ± 3.72	7.39 ± 1.85	< 0.001
Eccentric (%)	26(89.7%)	11(100%)	0.548
Enhancement (%)	4(13.8 %)	3(27.3%)	0.592
Intraplaque hemorrhage (%)	1(3.4%)	0(0.0%)	1.000
**Stenosis rate** (%)	43.30 ± 8.19	6.93 ± 6.84	< 0.001

The remodeling index was significantly different between the BAD and LI groups (0.97 ± 0.05 vs. 1.05 ± 0.01, *P* < 0.001, Table 6). Most BAD patients showed non-positive remodeling (93.1%), while the great majority of LI patients showed positive remodeling (81.8%).

**Table 6:** Artery remodeling modes of MCA atherosclerosis for BAD and LI in anterior circulation.

	**BAD (*n* = 29)**	**LI (*n* = 11)**	***P***
Remodeling index	0.97 ± 0.05	1.05 ± 0.01	< 0.001
Remodeling mode			< 0.001
Positive remodeling (%)	2(6.9%)	9(81.8%)	
Intermediate remodeling(%)	14(48.3%)	2(18.2%)	
Negative remodeling (%)	13(44.8%)	0(0.0%)	
Positive remodeling (%)	2(6.9%)	9(81.8%)	< 0.001

## Discussion

LI causes nearly a quarter of all ischemic stokes (1) playing an important role in cerebral vascular disease (18, 19). It used to be considered as small vessel lipohyalinosis blocked the distal end of the perforating artery. However, Chung JW et al (5) detected atherosclerotic plaques on HRMRI in the MCA and basilar artery in 60% of LI patients, confirming that atherosclerotic lesions in large arteries is an important underlying cause of LI. A considerable proportion of both BAD and LI cases are PAIs associated with large artery lesions, but the clinical manifestations and prognoses are distinctly different between these two groups. The results of our study show that this may be because of differences in artery wall morphology and plaque characteristics.

The rate of MCA plaques was significantly higher in the BAD group than in the LI group, indicating that the mechanisms of PAIs caused by carrier arterial disease were different in these two subtypes, which needed clinical attention. Small infarction zone should still be paid attention to exclude major arterial lesions and cannot be treated with the same general treatment strategy used in small artery disease.

The HRMRI imaging data showed that the BAD and LI groups had totally different artery remodeling modes. This may be key to explaining the distinct mechanisms underlying these conditions.

Arterial remodeling was first used in coronary stenosis by Glagov et al. (20). It describes the changes that occur in vessel area following artery atherosclerosis and can reveal positive remolding (PR) or negative remolding (NR). PR refers to the outward expansion of the vessel wall and the compensatory luminal dilatation that then occurs to reduce stenosis (21). NR refers to the contraction of the vessel wall and the consequential narrowing of the lumen, which increases stenosis (22).

Preliminary studies in coronary and carotid arteries have shown that PR is associated with unstable plaques. Plaques with PR tend to hemorrhage, rupture, and shed (23, 24), and their common pathological changes include inflammation and proliferation (25), which may be related to the increasing mobility of the plaque while the expansion of the outwall. NR was associated with stable plaques, and its pathological features include plaque calcification and sclerosis (25).

Research based on HRMRI has shown that the majority of patients with symptomatic MCA stenosis exhibit PR, while asymptomatic MCA stenosis patients show more NR. Shi et al. (26) found that there were more microemboli signals in a symptomatic MCA stenosis with PR, indicating that PR was associated with unstable plaques that tended to bleed, corrupt and fall off. It was also found that NR was associated with plaques with a larger fibrous component and a smaller lipid core (13), indicating that stable plaques are associated with NR.

**Figure 6:**
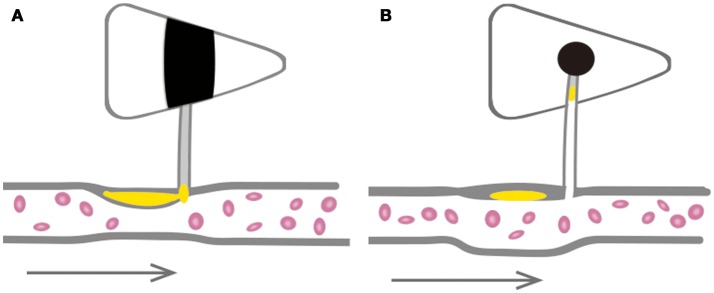
Schematic diagrams of large arteries involved in the pathogenesis of BAD and LI in anterior circulation. **(A)** BAD is probably associated with the occlusion of perforating artery with a relatively large and stable atherosclerosis plaque in great artery. **(B)** LI may be associated with the occlusion of distal branches of perforating artery by dropped embolus from relatively small and unstable atherosclerosis plaque in great artery.

In our study, we found that most BAD patients showed non-PR and had smaller lumen areas, a higher stenosis rate, a larger plaque area and a greater plaque burden. These results suggested that BAD is probably associated with the occlusion of perforating arteries by relatively large and stable atherosclerotic plaques originating in large arteries in the anterior circulation (Figure 6A). A thrombosis may gradually progress during the acute phase blocking an adjacent perforation, causing symptom progression. While the great majority of LI patients exhibited PR and had larger lumen areas, milder stenosis rates, smaller plaque areas, and lower plaque burdens, indicating that LI may be associated with the occlusion of the distal branches of perforating arteries by an embolus that originates in a relatively small and unstable atherosclerotic plaque in a large artery in the anterior circulation (Figure 6B). Therefore, LI often presents with mild clinical symptoms but can easily recur. The above connections are all based on imaging data obtained from a small group of patients. There is therefore potential for selection bias, and these results cannot be used to determine pathological causality.

Treatment for BAD emphasizes antithrombotic therapy in the acute phase and reducing plaque for prophylaxis, while treatment for LI should be focused on stabilizing the plaque to prevent recurrence. Clinically, patients with PAI need to complete a HRMRI examination and an assessment of artery remodeling and plaque size so that the appropriate treatment and prevention programs can be selected.

## Limitations and conclusions

Our study has several limitations. First, this study had a significantly limited sample size, and we recruited only symptomatic patients. Thus, we lacked control group for comparing vessel shape and plaque characteristics between symptomatic and asymptomatic patients. The onset of LI is usually mild and sometimes remains silent. Therefore, the rates at which BAD and LI patients present in hospitals vary significantly and will affect sampling from a population. Furthermore, this study enrolled only south Chinese patients, in whom the incidence of atherosclerotic infarction is 3–5 times higher than in westerners. Therefore, the results have limited applicability in the global population, and further study is needed. Second, our study showed that atherosclerotic features differ between BAD and LI. However, plaque vulnerability is a much more complicated issue. Both enhancement features and plaque composition are important characteristics that reflect plaque vulnerability. While we did analyze intraplaque hemorrhage and enhancement, these two indicators failed to show differences in our small sample size due to their low incidence. In addition, due to resolution and sequence limitations, we were unable to analyze differences in plaque composition, including fiber cap, lipid core and calcification, and obtaining these data would provide more information about these lesions. Therefore, interpreting the mechanisms underlying these two different stroke types requires further research. Third, the terms BAD and LI were originally defined based on pathological findings. However, due to a lack of pathological results, we used 15 mm and >2 slices (based on the majority previous studies) as the threshold. These parameters limited this study to the analysis of the pathogenesis of BAD and LI by imaging. Due to the limitations of the imaging techniques used in this study, we were only able to observe lesions in large arteries and did not examine any visual or pathological findings that might provide direct information regarding changes in branch arteries. Hence, further research is needed.

In conclusion, the results of our study confirm that while LI may share a connection with atherosclerotic plaques in large arteries, it occurs at a lower rate than that of BAD. Our HRMRI imaging data indicate that in the anterior circulation, BAD patients mainly showed relatively large and stable atherosclerotic plaques in large arteries and that LI patients mainly exhibited relatively small and unstable atherosclerotic plaques in large arteries.

Further studies performed using a multicenter design and comprehensive HRMRI sequences in intracranial arteries are needed to better explore the mechanisms underlying the different subtypes of PAI and for guiding clinical treatments.

## Author contributions

SL, ZD, and ZL: acquisition of data and drafing of the manuscript. SL, ZD, YW, and TJ: acquisition of data. ZK, ST, YS, and YZ: statistical analysis. SL, ZD, ZL, and ZY: revising the manuscript. ZL and YZ: conceiving the study and drafing of the manuscript.

### Conflict of interest statement

The authors declare that the research was conducted in the absence of any commercial or financial relationships that could be construed as a potential conflict of interest. The reviewer RH and handling editor declared their shared affiliation.
